# New Piezoceramic SrBi_2_Nb_2-2*x*_W*_x_*Sn*_x_*O_9_: Crystal Structure, Microstructure and Dielectric Properties

**DOI:** 10.3390/ma17184455

**Published:** 2024-09-11

**Authors:** Sergei V. Zubkov, Ivan A. Parinov, Alexander V. Nazarenko

**Affiliations:** 1 Research Institute of Physics, Southern Federal University, Rostov-on-Don 344090, Russia; svzubkov61@mail.ru (S.V.Z.); alex_v_nazarenko@mail.ru (A.V.N.); 2 I. I. Vorovich Mathematics, Mechanics and Computer Sciences Institute, Southern Federal University, Rostov-on-Don 344090, Russia; 3 Southern Scientific Center of the Russian Academy of Sciences, Rostov-on-Don 344090, Russia

**Keywords:** Aurivillius-Smolensky phase, SrBi_2_Nb_2-2*x*_W*_x_*Sn*_x_*O_9_ (*x* = 0.1, 0.2, 0.3, 0.4), activation energy *Ea*, Curie temperature *T_C_*, relative permittivity *ε*/*ε*_0_

## Abstract

By using the method of high-temperature solid-phase reaction, the new piezoceramic SrBi_2_Nb_2-2*x*_W*_x_*Sn*_x_*O_9_ was obtained, where partial substitution of niobium (Nb) atoms with Sn^4+^ and W^6+^ atoms in the compound SrBi_2_Nb_2_O_9_ occurred in the octahedra of the perovskite layer (*B*-position). X-ray diffraction investigations showed that these compounds are single-phase SrBi_2_Nb_2-2*x*_W*_x_*Sn*_x_*O_9_ (*x* = 0.1, 0.2) and two-phase SrBi_2_Nb_2-2*x*_W*_x_*Sn*_x_*O_9_ (*x* = 0.3, 0.4), but all of them had the structure of Aurivillius-Smolensky phases (ASPs) with close parameters of orthorhombic unit cells. It corresponded to the space group A21am. The temperature dependences of the relative permittivity ε/ε_0_ and the tangent of the dielectric loss angle tan *d* were defined at various frequencies. It was found that doping SrBi_2_Nb_2-2*x*_W*_x_*Sn*_x_*O_9_ (*x* = 0.1) improved the electrophysical properties of the compound: losses decreased, and the relative permittivity increased. This result was obtained for the first time. Moreover, a new result was obtained that indicated an improvement in the electrophysical properties of SrBi_2_Nb_2_O_9_ using the chemical element Sn (tin). This refutes the previously existing opinion about the impossibility to use Sn as a doping element.

## 1. Introduction

In 1949, while studying the Bi_2_O_3_–TiO_2_ system, B. Aurivillius established the formation of the oxide Bi_4_Ti_3_O_12_ with a perovskite-type structure [1,2,3]. After this, the group of G. Smolensky [4] established the ferroelectric properties of the compound PbBi_2_Nb_2_O_9_ from this class. This marked the beginning of a new stage in the search for and study of such compounds, which can thus be called Aurivillius-Smolensky phases (ASPs). The Aurivillius-Smolensky phases coincide with the currently generally accepted name–the Aurivillius phases. This term was first introduced by the authors in the paper [5]. Subsequently, Subbarao obtained about ten new ASPs, almost all of which were ferroelectrics [6,7]. ASPs are a large family of bismuth-containing layered perovskite-type compounds with the general chemical formula Bi_2_*A_m_*_−1_*B_m_*O_3*m*+3_.

The crystal structure of the Aurivillius-Smolensky phases consists of alternating (Bi_2_O_2_)^2+^ layers separated by perovskite-like (*A_m_*_−1_*B_m_*O_3*m*+1_)^2−^ layers, with the ions *A* having large radii Bi^3+^ [8], Ca^2+^, Gd^3+^ [9], Sr^2+^, Ba^2+^, Pb^2+^, Na^+^, K^+^, Y^3+^ [10], Ln^3+^, Nd^3+^ [11], Lu^3+^ [12] (lanthanides) and showing dodecahedral coordination. The *B*-positions in the oxygen octahedra are occupied by highly charged (≥3+) small-radius cations (Ti^4+^, Nb^5+^, Ta^5+^ [13], W^6+^ [14], Mo^6+^, Fe^3+^, Mn^4+^, Cr^3+^, Ga^3+^ [15], etc.). The number of perovskite layers (*A_m_*_−1_*B_m_*O_3*m*+1_)^2−^ located between the fluorite-like layers (Bi_2_O_2_)^2+^ along the pseudotetragonal axis *c* (001) determines the value of *m*, which can be an integer or half-integer in the interval of *m* = 1−6.

Atom substitutions in the *A*- and *B*-positions define significantly the electrical and physical properties of ASPs. Considerable alterations in the permittivity and electrical conductivity occur; moreover, the Curie temperature *T*_C_ can also change within broad limits. Thus, the investigation of cation-substituted ASPs compounds is very important for design of materials used in different technical applications (for example, solid-state gas sensors, non-volatile memory elements, solid-state displays, optical switches, and storage devices). The replacement of Nb^5+^ with a metal cation in the structure of bismuth titanate oxide of the ASPs solid solution family requires compliance with certain criteria.

The first consideration is the balance of the electron charges. Although the principle of equality allows only ions with a charge of 5+ to be exchanged, by mixing impurities, cations with different oxidation states can be used. For example, doping can involve a mixture of 4+ and 6+ ions or various substitutions of *A*-cation to balance the available charges.

Other treatment in doping relates to the sizes of the ionic radii. The pseudo-perovskite layer (PPL) has a tolerance factor *t* that belongs to a smaller interval than that of standard perovskite (*t* = 0.81–0.93 instead of *t* = 0.77–1.0124, respectively). This determines that the ionic radius of the cation in the oxygen octahedron should be in the interval of 0.58–0.65 Å. The lower boundary of this interval is defined by the loss of stability of the pseudo-perovskite structure due to the internal deformation that occurs when trying to dope smaller cations. At the same time, the upper boundary is a result of the size mismatch between the pseudo-perovskite (*A_m_*_−1_*B_m_*O_3*m*+1_)^2−^ and (Bi_2_O_2_)^2+^ layers; if the layers cannot align, a steady structure cannot be formed [16]. This feature is evident for Ge^4+^, whose ionic radius of 0.53 Å is outside the substitution interval due to a very small ionic radius. Other examples are related to Sn^4+^, Hf^4+^ and Zr^4+^ with ionic radii of 0.69 Å, 0.71 Å, and 0.72 Å, respectively, placing them outside the above-mentioned (0.58–0.65 Å) interval, but at the opposite end of the scale in this case.

The crystal structure of layered perovskites cannot vary freely by doping, because doping is restricted by the (Bi_2_O_2_)^2+^ interlayer. When Sr^2+^ (~143 pm) is replaced in the *A*-position by a large Ba^2+^ ion (~160 pm), the Curie temperature *T*_C_ decreases. When a small Ca^2+^ ion (~136 pm) replaces the Sr^2+^ ion, the value of *T*_C_ increases. This can be explained by the fact that more space is required to introduce a large ion into the *A*-position, despite the increase in the cell parameters. Due to this, the mobility of the oxygen octahedron ions diminishes together with the Curie temperature. Thus, according to [17], the value of *T*_C_ for the Bi_2_Sr_1−*x*_Ca*_x_*Nb_2_O_9_ (CBN) and Bi_2_Sr_1−*x*_Ba*_x_*Nb_2_O_9_ (BBN) compounds is equal to ~620 °C and ~200 °C, respectively. It explains relation of the Curie temperature to ionic polarization, which is determined by structural distortions. The large difference between the parameters *a* and *b* for CBN in comparison with BBN and SrBi_2_Nb_2_O_9_ (SBN) defines a higher Curie temperature *T*_C_ [18,19,20,21,22,23,24,25,26,27].

Piezoceramic SrBi_2_Nb_2_O_9_ is one of the most prospective compounds for creating ferroelectric memory elements, since it is very resistant to external influences [18]. The effect of ion substitution in perovskite cells, both in the crystallographic *A*-position and in the *B*-position, has been the subject of many studies [22,28,29,30,31,32].

The results of the influence of doping on the microstructure, electrical, and physical properties of layered SBN structures are present in [33], where Bi_2−*x*_Te*_x_*Sr_1−*x*_K*_x_*Nb_2_O_9_ with 0 ≤ *x* ≤ 0.25 were investigated. It was defined that the values of *T*_C_ decrease almost linearly with the increase in the parameter *γ* = (*abc*)^1/3^, that is, the larger the volumes of the pseudo octahedra, the lower the Curie temperature of the studied materials.

Moreover, in [33] the replacement of Sr^2+^ ions by Ca^2+^ and Ba^2+^ ions in the *A*-position and Nb^5+^ ions by V^5+^ ions in the *B*-position up to 30 at. % was investigated. It was established that the lattice constants, dielectric, and electrical properties of SBN ceramics significantly depend on the type and number of doping atoms. It was established that doping with vanadium has a considerable effect on the dielectric and ferroelectric properties of the doped structures [34,35,36,37,38]. In particular, the residual polarization of the SBN ferroelectric, when doped with 10 at. % vanadium, grew from ~2.8 μC/cm^2^ to ~8 μC/cm^2^, and the coercive field diminished from ~63 kV/cm to ~50 kV/cm. It was also shown that doping of the initial composition with Ca^−^ and V^−^ ions increases the value of *T*_C_ with the growth of doping level. On the contrary, doping the initial composition with V-ions decreases the Curie temperature *T*_C_, and the permittivity at *T*_C_ increases almost twice at 10 at. % of V compared to SBN. The study of the permittivity of SrBi_2_Nb_2_O_9_ doped with Mo and Cr and its frequency dependence showed that in the case of both types of doping, it increased with an increasing concentration of impurity atoms, and the sharpest increase was observed in the region of low concentrations.

In this study, the possibilities of doping the bilayer compound SrBi_2_Nb_2_O_9_ with Sn^4+^ and W^6+^ cations are considered. The innovation and significance of this paper are explained by the fact that for the first time it refutes the previously existing opinion about the impossibility to use Sn as a doping element.

The remaining parts of this paper are organized as follows: Section 2 describes in detail manufacturing of the SrBi_2_Nb_2-2*x*_W*_x_*Sn*_x_*O_9_ compounds and the experimental methods of their study. Section 3 presents the experimental powder X-ray diffraction patterns of the studied solid solutions SrBi_2_Nb_2-2*x*_W*_x_*Sn*_x_*O_9_ (*x* = 0.1, 0.2, 0.3, 0.4), their microstructure, crystalline structure, dielectric properties, results of Mössbauer studies, and activation energy. The conclusions are summarized in Section 4.

## 2. Manufacturing and Experimental Methods

Polycrystalline samples of SrBi_2_Nb_2-2x_W_x_Sn_x_O_9_ (*x* = 0.1, 0.2, 0.3, 0.4) of the ASPs series were synthesized by solid-phase reaction of the corresponding oxides Bi_2_O_3_, Nb_2_O_5_, W_2_O_5_, SnO_2_, and strontium carbonate SrCO_3_. The oxides and carbonate used were of extra pure degree, meaning the reagents contained at least 99% of the main substance, and the remaining 1% or less of impurities did not interfere with their use for analytical purposes. After weighing according to the stoichiometric composition and thoroughly grinding the initial substances with the addition of ethyl alcohol, the pressed specimens were subjected to calcination at a temperature of 840 °C for 4 h. The temperature of 840 °C was reached within 1 h. The specimens were subjected to fire in a laboratory furnace in air. They were then crushed, repeatedly ground, and pressed into tablets with a diameter of 10 mm and a thickness of 1.0–1.5 mm, followed by final sintering at a temperature of 1150 °C for 3 h (the temperature of 1150 °C was reached within 2.5 h).

A Rigaku Ultima IV diffractometer with a Cu X-ray tube was used to obtain X-ray diffraction pattern. A Ni-filter was used to isolate the Cu *K*α_1_,α_2_ radiation from the total spectrum. The X-ray diffraction pattern was measured in the 2θ angle interval from 10° to 60° using a scanning step of 0.02° and an exposure (intensity recording time) of 4 s per point. The PCW 2.4 software was used to analyze the X-ray diffraction pattern profile, to determine the positions of lines, their indexation (*hkl*), and to refine the unit cell parameters [39].

In experimental studies of the relative permittivity and electrical conductivity, electrodes were applied to the flat surfaces of the ASPs specimens using Ag paste, annealed at a temperature of 800 °C for 30 min. The specimens had disk form with a diameter of 10 mm and a thickness of about 1.5 mm. An E7-20 immittance meter was used to measure the temperature and frequency dependences of the dielectric characteristics (relative permittivity and dielectric loss tangent) in the frequency interval from 100 kHz to 1 MHz and in the temperature interval from room temperature to 750 °C. The heating rate in the experiment was 5 °C per 2 min. The measurements were performed with an interval of 5 min.

A Carl Zeiss EVO 40 scanning electron microscope (Zeiss, Oberkochen, Germany) was used to obtain microstructure images. The study was performed on the cross-section chips of the manufactured ceramics. Grain blurring and multiple charge accumulation effects were observed in the absence of an additional conductive layer. Therefore, the conductive layer was deposited with the help of an SC7620 Mini Sputter Coater magnetron sputtering unit to analyze the chip surface (QUORUM, Laughton, East Sussex, UK). Before sputtering, the specimens had not been preliminarily subjected to mechanical treatment. The investigation was performed in the high accelerating voltage mode (EHT = 20 kV). To increase the resolution, the probe current was set at *I*_probe_ = 55 pA, and the working distance WD = 8–9 mm. Grain size analysis was performed using “Gwyddion”, specialized free and open-source data analysis software (Gwyddion v. 2.61), covered by GNU General Public License by David Nečas and Petr Klapetek [40]. Gwyddion provides many data processing functions, including all those for the standard statistical characterization. One of the many possibilities is that Gwyddion allows, based on the metadata of SEM image, the measurement of linear sizes and their accumulation in an array with the possibility of further copying and mathematical processing. To determine the linear sizes of particles, the largest diagonal of the visible part of the grain on the chip was selected. Submicron and micron grain sizes were measured and analyzed separately. The submicron size array was ranked with intervals of 0.1–0.2 μm, while the micron size array was ranked with intervals of 0.5–1.0 μm.

## 3. Results and Discussion

### 3.1. Diffraction

**Figure 1:**
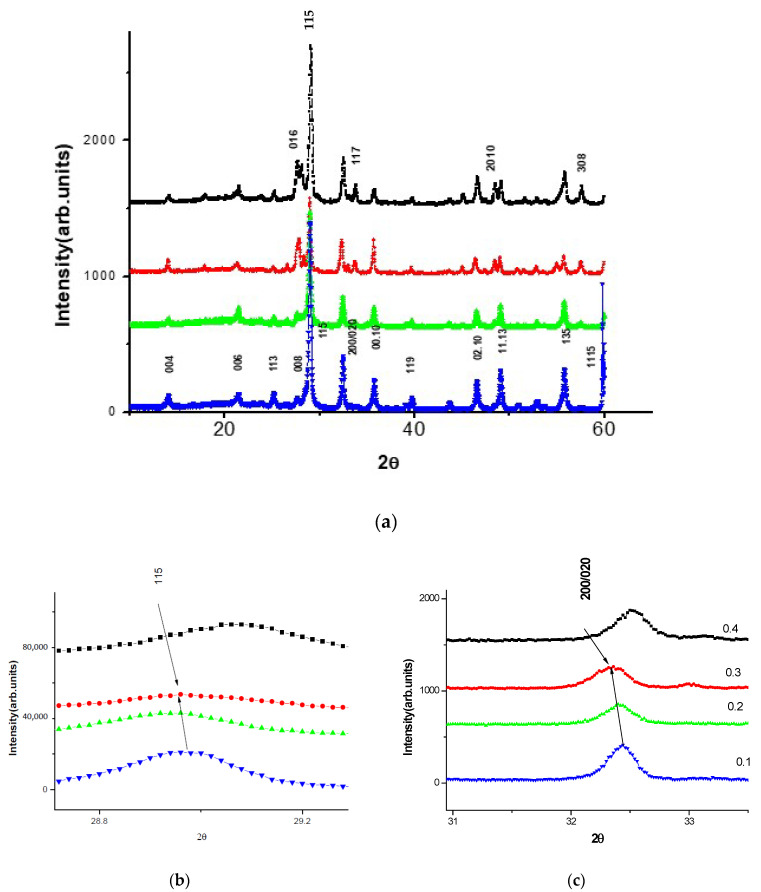
Test X-ray diffraction patterns of SrBi_2_Nb_2-2*x*_W*_x_*Sn*_x_*O_9_ (*x* = 0.1, 0.2, 0.3, 0.4) compounds: (**a**) general picture; (**b**) fragment of the main reflection 115; (**c**) fragment of the evolution of the 200/020 line. 4 colored lines correspond to *x* = 0.1, 0.2, 0.3, 0.4. It is pointed in (**c**) for all three figures. The arrows show reflexes pointed above them.

Figure 1 demonstrates the experimental powder X-ray diffraction patterns of the investigated solid solutions SrBi_2_Nb_2-2*x*_W*_x_*Sn*_x_*O_9_ (*x* = 0.1, 0.2, 0.3, 0.4), with indexation of the most important reflections. It was established that the synthesized solid solutions crystallize in the orthorhombic system with the space group of the unit cell A21am and correspond to the ASPs with *m* = 2. It is possible to observe the evolution of the intensity of some lines, as well as the appearance and disappearance of others in SrBi_2_Nb_2-2*x*_W*_x_*Sn*_x_*0_9_ (*x* = 0.3, 0.4).

The tendency of the main reflex 115 to shift towards larger angles is clearly expressed for concentrations of 0.1 and 0.2, and to a lesser extent, for *x* = 0.3. This tendency is also characteristic of reflex 200. It was also established that the main reflection 115 (112*n* + 1) SrBi_2_Nb_2-2*x*_W*_x_*Sn*_x_*O_9_ (*x* = 0.1, 0.2, 0.3) in Figure 1b and the parameter (200) shifts towards smaller angles in Figure 1c for the same concentration of W*_x_*Sn*_x_*.

In this study, the niobium ion is replaced by a tin ion, which has a larger ionic radius, leading to an increase in lattice parameters. In turn, an increase in lattice parameters leads to a shift in the reflections towards smaller angles, observed for concentrations of *x* = 0.1, 0.2, and to a lesser extent, *x* = 0.3. It can be assumed that the solubility of tin ends approximately in the region of *x* = 0.2.

According to X-ray diffraction results [41], the parameters of the crystal lattice were found, based on which orthorhombic and tetragonal distortions were calculated; these are presented in Table 1.

### 3.2. Microstructure

**Figure 2:**
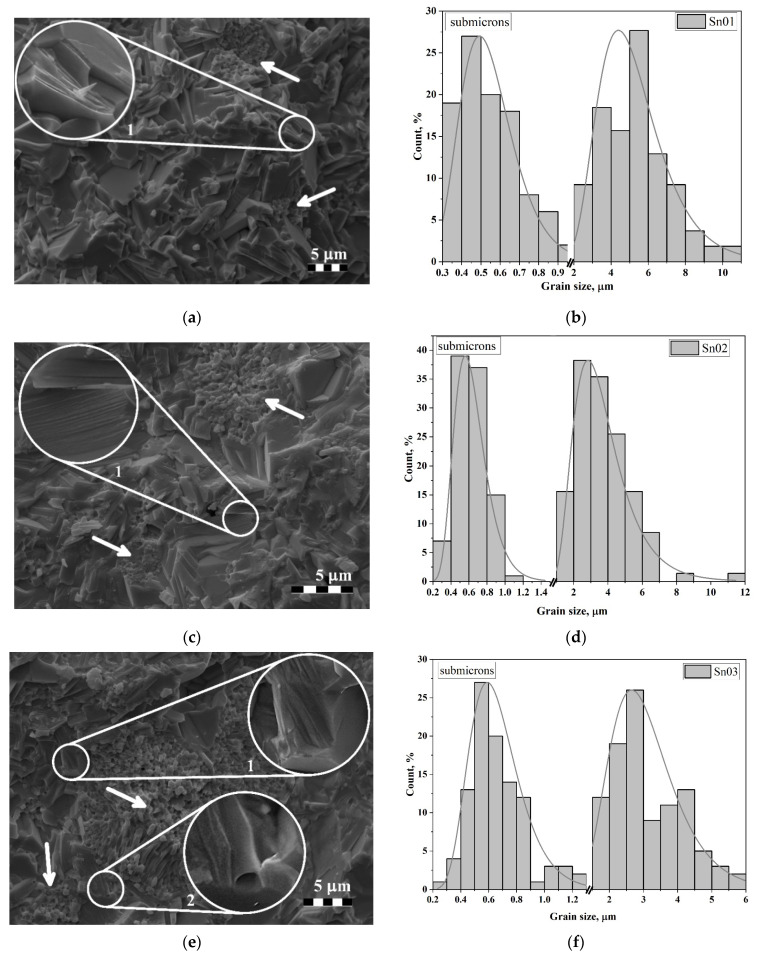
Surface areas of cleavages at Sn concentrations: (**a**) *x* = 0.1, (**c**) *x* = 0.2, (**e**) *x* = 0.3; histograms of grain size distributions at Sn concentrations: (**b**) *x* = 0.1, (**d**) *x* = 0.2, (**f**) *x* = 0.3. Increased circle areas show increased imagines of small circle areas. Arrows show locations these areas.

An analysis of cross-section chips images demonstrated that the average grain size diminishes from ~5.4 μm to ~1.2 μm with an increase in Sn concentration. At *x* > 0.3, in contrast to the samples with lower concentrations of Sn, the microstructure becomes less dense with the present of numerous pores, which are apparently of a technological nature (that is, their size, shape, and nature of arrangement do not correspond to the size and shape of the grains themselves). Up to *x* = 0.3, the chip mainly passes along grains that are tightly packed and have a lamellar shape with well-defined boundaries. This indicates anisotropic crystallite growth, characteristic of ASPs. The few presented pores are of a diffusion character. The grains are grown together randomly. Their internal structure is non-uniform and has a layered structure, which is obvious on the “sloped” cleavages of the grains themselves (see Figure 2a,c,e insets 1). In addition, there are large clusters of prismatic grains of submicron size with rounded edges present in the image (see Figure 2a,c,e arrows). At low concentrations (*x* = 0.1), such clusters are local, and the crystallites are somewhat smaller in size than at higher concentrations (*x* = 0.3). In general, the growth of grains in size is about 20%.

With increasing concentration to *x* = 0.3, the amount of the submicron phase becomes so large that some interesting features appear. On the crossed-section surface, where grains are clustered, elongated crystallites appear alongside diffusion pores. Moreover, the presence of pores in the form of channels (Figure 2e, insert 2) is noteworthy. It can be assumed that these noted diffusion pores are nothing more than intergranular channels, and the elongated parts are images of the boundaries of these channels along which cleavage passed. In this regard, it can be concluded that a similar pattern takes place at concentrations of *x* = 0.1–0.2, but due to the smaller sizes of both the crystallites themselves and the areas of their clusters, these channels are less pronounced, which does not allow them to be observed explicitly.

The presence of two clearly separated grain fractions gave a basis for analyzing their sizes separately. For illustration and comparison purposes, the histograms were plotted on the same axes (see Figure 2). It is evident that each distribution has a lognormal character, although for *x* = 0.1 and 0.3, there are some anomalies that can be associated with many semi-closed crystallites, the true sizes of which cannot be determined. The average sizes of crystallites in the large fraction are about 5.4 μm, 3.8 μm, and 3.1 μm, and in the submicron fraction about 0.55 μm, 0.63 μm, and 0.68 μm for *x* = 0.1, 0.2 and 0.3, respectively. It should be additionally noted that with increasing Sn concentration, the size of lamellar relatively large grains decreases, while that of prismatic submicron grains increases.

**Figure 3:**
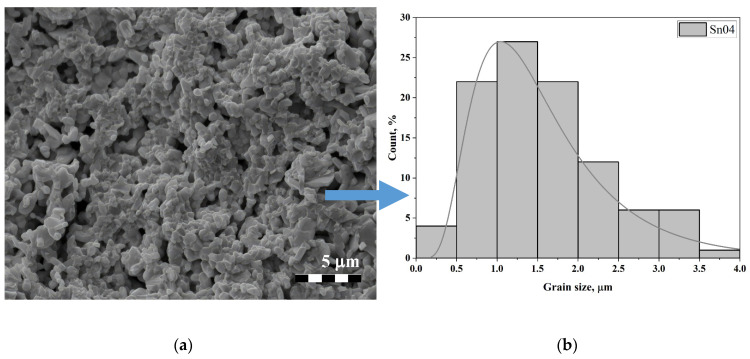
(**a**) Microstructure of cleavage surface area of material with concentration of Sn equal to *x* = 0.4 and (**b**) histogram of grain size distribution.

At concentrations *x* > 0.3, the chip occurs along the grain boundaries. As noted earlier, there are numerous pores, the nature of which can be explained by an increase in the pyrochlore phase and the replacement of bismuth ions by Sn ions in the bismuth-oxygen layer, which leads to decreased stability of the crystal structure [42]. At *x* = 0.4, maximum mixing of phase states is observed because of trends toward an increase in the size of prismatic grains (previously submicron in size) and a decrease in the size of lamellar grains (previously relatively large) (see Figure 3a). At this concentration, they become commensurate. Therefore, the average crystallite size decreases by approximately 50% compared to *x* = 0.3, to about 1.58 μm. In this case, the lamellar grains are located as if “in the mass” of prismatic grains, which form dense conglomerates. Prismatic grains apparently demonstrate an increase in the pyrochlore phase compared to the ASPs. The distribution histogram also has a lognormal, almost classical character (see Figure 3b).

Considering the grain sizes, the spread of lengths is quite big, ranging from 0.2 to 12 µm. This can be explained by both the existing of submicron particles and the chaotic arrangement of plane grains in the ceramic volume. In the latter case, the chipping of the grain itself can occur at a random place, considering the natural spread in grain sizes, the chipped projections can have a continuous spectrum of length.

The crystallite thickness fluctuates in the interval of 1–3 µm. This also corresponds to ASP-type ceramics. The nature of the grain arrangement is such that they stick together perpendicular to the plane (*ab*), creating “stacks” of thin (~50–400 nm) plates (see Figure 2, highlighted areas).

### 3.3. Crystalline Structure

As noted above, Table 1 presents the unit cell parameters of the obtained samples of SrBi_2_Nb_2-2*x*_W*_x_*Sn*_x_*O_9_ (*x* = 0.1, 0.2, 0.3, 0.4) solid solutions, as well as the values of orthorhombic (*δb*_0_) and tetragonal (*δc′*) strains, the average tetragonal period (*a_t_*), the tolerance factor (*t*), and the average thickness of one perovskite layer (*c′*). Here, *c′* = 3*c*_0_/(8 + 6*m*) is the thickness of a single perovskite-like layer; *a_t_ =* (*a*_0_ *+ b*_0_)/2^3/2^ is the average value of the tetragonal period; *a*_0_, *b*_0_, *c*_0_ are the lattice periods; *δc′ =* (*c′–a_t_*)/*a_t_* is the deflection of the cell from the cubic shape; *δb*_0_ *=* (*b*_0*−*_*a*_0_)/*a*_0_ is the orthorhombic strain [43].

The parameters of the crystal cells of the synthesized compounds SrBi_2_Nb_2-2*x*_W*_x_*Sn*_x_*O_9_ (*x* = 0.1, 0.2, 0.3, 0.4) are close to the previously determined characteristics of SrBi_2_Nb_2_O_9_: *a* = 5.55 Å, *b* = 5.48 Å, *c* = 25.261 Å [44]. For the synthesized compounds, the tolerance factor *t* was calculated [42] as a geometric criterion determining the degree of stability and distortion of the crystal structure:

(1) $$t=(R_{A}+R_{O})/[\sqrt{2}(R_{B}+R_{O})],$$

where *R_A_* and *R_B_* are the cation radii in positions *A* and *B*, respectively; *R*_O_ is the ionic radius of oxygen.

The values of the tolerance factor *t* for the studied sample are given in Table 2. In the present work, the tolerance factor was defined by using the Shannon ionic radii [45] for the corresponding coordination numbers (CNs) O^2−^ (CN = 6), *R*_O_ = 1.40 Å; Nb^5+^ (CN = 6) *R*_Nb_^5+^ = 0.6 Å. Shannon did not provide the radius of Bi^3+^ ion for coordination with CN = 12. Therefore, its value was calculated by using the radius of ion with CN = 8 (*R*_Bi_^3+^ = 1.17 Å), multiplied by the approximation factor 1.179, then for Bi^3+^ (CN = 12) we defined *R*_Bi_^3+^ = 1.38Å.

### 3.4. Dielectric Properties

**Figure 4:**
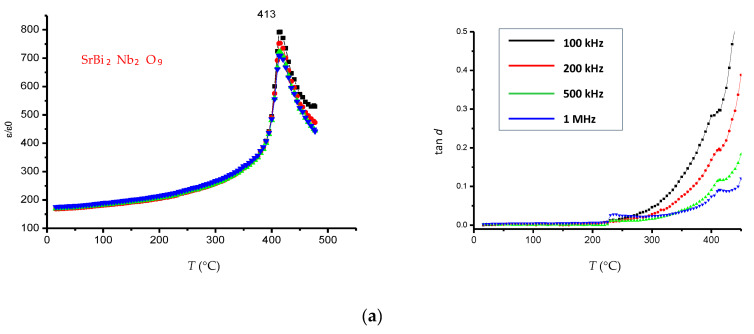

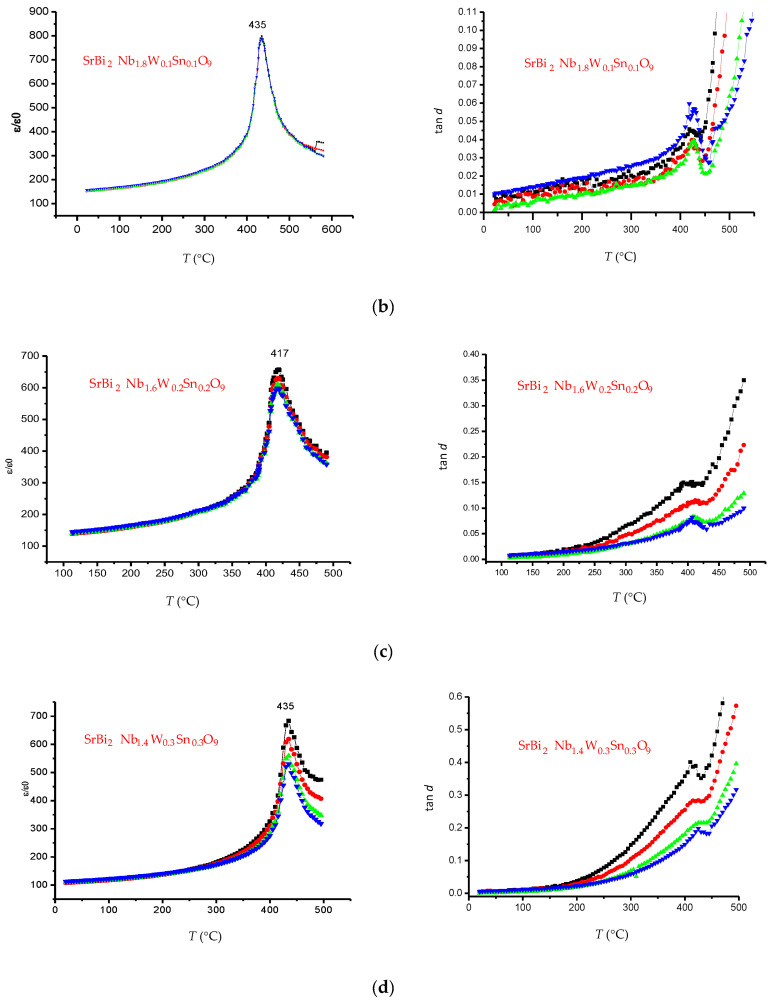

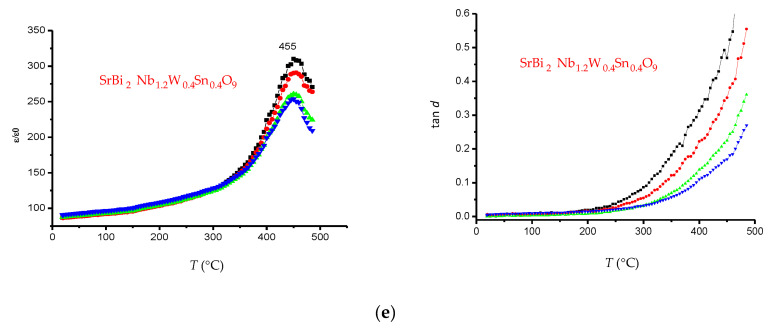
Relative permittivity *ε*/*ε*_0_ (**left**) and dielectric loss tangent tan *d* (**right**) vs. temperature for solid solutions SrBi_2_Nb_2-2*x*_W*_x_*Sn*_x_*O_9_: (**a**) *x* = 0.0; (**b**) *x* = 0.1; (**c**) *x* = 0.2; (**d**) *x* = 0.3; (**e**) *x* = 0.4 at a frequency range from 100 kHz to 1 MHz.

Figure 4 demonstrates the temperature dependences of the relative permittivity *ε*/*ε*_0_ and the dielectric loss tangent tan *d* for SrBi_2_Nb_2-2*x*_W*_x_*Sn*_x_*O_9_ (*x* = 0.1, 0.2, 0.3, 0.4) across a frequency range from 100 kHz to 1 MHz.

The maximum permittivity, corresponding to the transition from ferroelectric to paraelectric phase (*T*_C_), is obvious for all compounds in the synthesized series of solid solutions of the ASPs SrBi_2_Nb_2-2*x*_W*_x_*Sn*_x_*O_9_ (*x* = 0.0, 0.1, 0.2, 0.3, 0.4) (at frequency range from 100 kHz to 1 MHz). The peak value of the relative permittivity diminishes with the increase in Sn and W concentration, which correlates with a reduction in grain size. The Curie temperature demonstrates a rise with an increasing concentration of W*_x_*Sn*_x_*. The values of the dielectric loss tangent for SrBi_2_Nb_2-2*x*_W*_x_*Sn*_x_*O_9_ (*x* = 0.1, 0.2, 0.3, 0.4) are approximately the same. For SrBi_2_Nb_1.8_W_0.1_Sn_0.1_O_9,_ the dielectric loss tangent decreases by 10-fold, and for SrBi_2_Nb_1.6_W_0.2_Sn_0.2_O_9_, it decreases by 2-fold compared to SrBi_2_Nb_2_O_9_. With the increase in temperature, the dielectric loss increases, demonstrating a clearly defined maximum for SrBi_2_Nb_2-2*x*_W*_x_*Sn*_x_*O_9_ (*x* = 0.0, 0.1, 0.2, 0.3) at all measured frequencies, and then decreases quickly. The minimum of dielectric loss usually precedes the peak of permittivity by 5 °C, but this is not always.

It is obvious that at Sn and W concentrations within the interval of *x* = 0.1–0.2, the dielectric properties of the synthesized compounds improve, likely due to a decrease in oxygen vacancies (composition defects). This result coincides with previously obtained results by doping Bi_3_Ti_1-*x*_Sn*_x_*NbO_9_ with Sn ions at a concentration of *x* = 0.1 [41]. This makes it possible to assert that vacancies are filled with Sn ions, and the most effective concentrations for improving the dielectric properties by doping with Sn ions lie within the interval of 0.1 < *x* < 0.2. A further increase in Sn concentration at *x* > 0.2, as follows from the microstructural analysis, enlarges prismatic grains and reduces lamellar grains, corresponding to the ASPs. The prismatic grains are usually associated with the appearance of the pyrochlore phase. A decrease in defects (a decrease in oxygen vacancies) leads to reduced transfer of matter and, therefore, reduces the size of lamellar grains. As is known, the growth of lamellar grains has an anisotropic character and occurs in the *ab*-plane.

### 3.5. Mössbauer Studies

**Figure 5:**
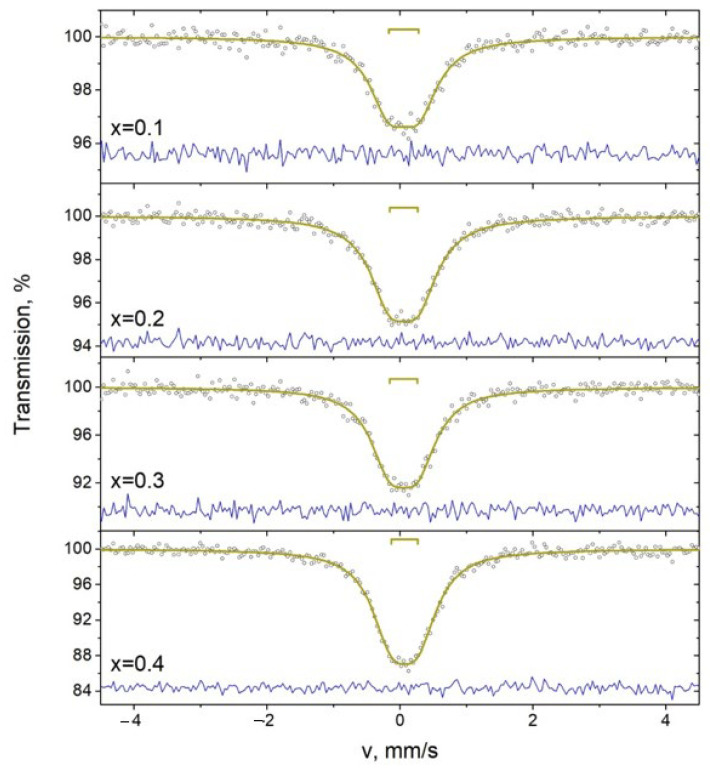
Mössbauer spectra 119Sn of SrBi_2_Nb_2-2*x*_W*_x_*Sn*_x_*O_9_ (*x* = 0.1, 0.2, 0.3, 0.4) specimens at room temperature. Circles present experimental results and lines present average experimental results.

Mössbauer spectrometer MS-1104Em (Southern Federal University, Rostov-on-Don, Russia), operating in the non-constant acceleration mode, was used to the Mössbauer studies, which were performed in absorption geometry. As a gamma-ray source, 119Sn in CaSnO_3_ was used. The spectra were calibrated with help of the spectra from BaSnO_3_ and α-Fe standards. The analysis of the Mössbauer spectra was performed using the SpectrRelax software (Moscow State University, Moscow, Russia, 1st version). The Mössbauer spectra of 119Sn in the SrBi_2_Nb_2-2*x*_W*_x_*Sn*_x_*O_9_ (*x* = 0.1, 0.2, 0.3, 0.4) compounds are present in Figure 5 [41].

The spectra are paramagnetic doublets, with the characteristics given in Table 3. The isomer shift values of the doublets correspond to Sn^4+^ in the oxygen octahedron. The presence of quadrupole splitting indicates that the symmetry of Sn^4+^ is lower than cubic. This decrease in symmetry may be due to lattice distortions or disordering of the composition.

### 3.6. Activation Energy

Activation energy *E_a_* was calculated using the Arrhenius equation:

*σ* = (*A*/*T*)exp[−*E_a_*/(*kT*)], (2)

where *σ* is the electrical conductivity, *k* is the Boltzmann constant, *A* is a constant, and *E_a_* is the activation energy.

**Figure 6:**
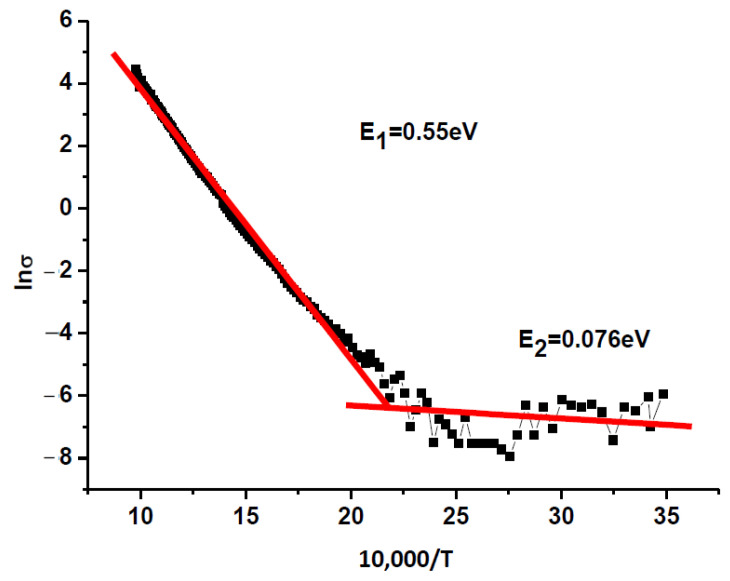
Dependence of ln *σ* on 10,000/*T* for SrBi_2_Nb_1.8_W_0.1_Sn_0.1_O_9_ at a frequency of 100 kHz. Red lines present approximated results, shown by points.

A calculated dependence of ln *σ* on 1/*T* (at a frequency of 100 kHz), used to define the activation energy *E_a_*, is demonstrated in Figure 6 for the SrBi_2_Nb_2-2*x*_W*_x_*Sn*_x_*O_9_ (*x* = 0.1, 0.2, 0.3, 0.4) compounds. These compounds show two temperature ranges where the values of activation energy *E_a_* differ considerably. In the low-temperature range, the electrical conductivity is defined mainly by impurity defects. The high-temperature range determines the intrinsic conductivity. For the SrBi_2_Nb_2-2*x*_W*_x_*Sn*_x_*O_9_ (*x* = 0.1, 0.2, 0.3, 0.4) compounds, the region with expressed impurity conductivity is observed in the temperature interval from 20 °C to 230 °C.

Figure 6 demonstrates a typical dependence of the conductivity logarithm on temperature. For the synthesized solid solutions SrBi_2_Nb_2-2*x*_W*_x_*Sn*_x_*O_9_ (*x* = 0.1, 0.2, 0.3, 0.4), the activation energy *E*_2_ in the high-temperature part remains almost unchanged, and in the low-temperature region, the activation energy *E*_2_ increases with the growth of W*_x_*Sn*_x_* (*x* = 0.2, 0.3), as follows from Table 2.

## 4. Conclusions

Obviously, the compounds consisting of Aurivillius-Smolensky phases has a great impact on the development and improvement of materials for different technical applications (for example, solid-state gas sensors, non-volatile memory elements, solid-state displays, optical switches, and storage devices).

A new compound, SrBi_2_Nb_2-2*x*_W*_x_*Sn*_x_*O_9_ (*x* = 0.0, 0.1, 0.2, 0.3, 0.4), of the ASP family was synthesized in this investigation. X-ray diffraction data showed that all solid solutions have a structure close to the Aurivillius-Smolensky phases with an orthorhombic unit cell (space group A21am). With an increase in the concentration of W*_x_*Sn_x_, the evolution of reflexes and the appearance of new lines in the diffraction pattern were observed. It was found that with an increase in W*_x_*Sn_x_, the grain size decreased, and, accordingly, the value of the relative permittivity also decreased. For the entire synthesized series of SrBi_2_Nb_2-2*x*_W*_x_*Sn*_x_*O_9_ (*x* = 0.0, 0.1, 0.2, 0.3, 0.4) the Curie temperature *T*_C_ increased from 417 °C to 455 °C with increasing W*_x_*Sn_x_. Microstructural analysis showed that with increasing W*_x_*Sn_x_ there was a decrease in lamellar grains, corresponding to the ASPs, and an increase in the prismatic grains, corresponding to the pyrochlore phase. At the concentration of W*_x_*Sn_x_ (*x* = 0.1), the tangent of the dielectric loss angle decreased 10-fold compared to SrBi_2_Nb_2_O_9_, which allows using Sn to improve the ferroelectric properties of ASPs compounds.

For the first time, a result was obtained to improve the electrophysical properties of SrBi_2_Nb_2_O_9_ using the chemical element Sn. This refutes the previously existing opinion about the impossibility to use Sn as a doping element.

## Figures and Tables

**Table 1:** Unit cell parameters (*a*_0_, *b*_0_, *c_0_*, *V*), tetragonal period parameter (*a_t_*), octahedron height along the *c*-axis (*c*′), deviation from cubic shape (*δc*′), rhombic distortion (*δb*_0_).

Compounds	*a*_0_, Å	*b*_0_, Å	*c*_0_, Å	*V*, Å^3^	*a_t_*,%	*c′*, Å	*δc′*,%	δ*b*_0_,%
SrBi_2_Nb_2_0_9_	5.55	5.48	25.261	768.23	3.899	3.79	−2.8	−1.2
SrBi_2_Nb_1.8_W_0.1_Sn_0.1_O_9_	5.49	5.45	25.16	754.20	3.87	3.77	−2.52	−0.8
SrBi_2_Nb_1.6_W_0.2_Sn_0.2_O_9_	5.52	5.51	25.16	766.18	3.90	3.77	−2.52	−0.2
SrBi_2_Nb_1.4_W_0.3_Sn_0.3_O_9_	5.50	5.57	25.34	776.13	3.91	3.80	−2.87	1.27
SrBi_2_Nb_1.2_W_0.4_Sn_0.4_O_9_	5.53	5.51	25.16	766.78	3.90	3.77	−2.87	−0.2

**Table 2:** Dielectric characteristics of SrBi_2_Nb_2-2*x*_W*_x_*Sn*_x_*O_9_ (*x* = 0.1, 0.2, 0.3, 0.4): Curie temperature *T*_C_, tolerance factor *t*, relative permittivity *ε*/*ε*_0,_ and activation energy. *E_a_* in high-temperature and low-temperature regions.

Compound	*T_C_*_,_ °C	*t*	*ε*/*ε*_0_ (at 100 kHz)	*E*_1_/*E*_2_, eV
SrBi_2_Nb_2_0_9_	413	0.9778	792	0.67/0.06
SrBi_2_Nb_1.8_W_0.1_Sn_0.1_O_9_	435	0.97	850	0.55/0.076
SrBi_2_Nb_1.6_W_0.2_Sn_0.2_O_9_	417	0.97	660	0.49/0.26
SrBi_2_Nb_1.4_W_0.3_Sn_0.3_O_9_	435	0.97	690	0.54/0.13
SrBi_2_Nb_1.2_W_0.4_Sn_0.4_O_9_	455	0.97	308	0.52/0.093

**Table 3:** Characteristics of Mössbauer spectra 119Sn of SrBi_2_Nb_2-2*x*_W*_x_*Sn*_x_*O_9_ (*x* = 0.1, 0.2, 0.3, 0.4) specimens at room temperature.

x	Component	δ ± 0.02, mm/s	Δ ± 0.02, mm/s	Γ ± 0.02, mm/s	A ± 1, %
0.1	D	0.06	0.45	0.73	100
0.2	D	0.06	0.43	0.72	100
0.3	D	0.05	0.48	0.73	100
0.4	D	0.07	0.39	0.73	100

*δ* is the isomer shift, Δ is the quadrupole splitting, Γ is the line width, A is the component area.

## Data Availability

The original contributions presented in the study are included in the article, further inquiries can be directed to the corresponding author.
